# Ultrasonographic characteristics of medullary thyroid carcinoma: a comparison with papillary thyroid carcinoma

**DOI:** 10.18632/oncotarget.15897

**Published:** 2017-03-04

**Authors:** Mei-juan Liu, Zhong-feng Liu, Yuan-yuan Hou, Yan-Ming Men, Yu-Xi Zhang, Ling-Yun Gao, Hao Liu

**Affiliations:** ^1^ Department of Ultrasound, The Affiliated Yantai Yuhuangding Hospital of Qingdao University, Yantai, Shandong, China; ^2^ Department of Ultrasound, Yantai Affiliated Hospital of Binzhou Medical University, Yantai, Shandong, China; ^3^ Department of Obstetrics and Gynecology, The Affiliated Yantai Yuhuangding Hospital of Qingdao University, Yantai, Shandong, China; ^4^ Department of Ultrasound, Yantai Hospital of Traditional Chinese Medicine, Yantai, Shandong, China

**Keywords:** medullary thyroid carcinoma, papillary thyroid carcinoma, ultrasound, diagnosis

## Abstract

This study was designed to explore differences in the ultrasonographic characteristics of medullary thyroid carcinoma (MTC) and papillary thyroid carcinoma (PTC). This study included 35 cases of MTC and 96 cases of PTC that were surgically and pathologically confirmed. Preoperative ultrasound images were retrospectively reviewed by two physicians (with 5 years’ experience in thyroid ultrasound) under the premise of unknown pathological results. Various ultrasonic features of nodules were assessed objectively. The clinical features of components were determined by other physicians. Age, sex, unilateral or bilateral involvement of thyroid gland, lesion size, margin, shape, echogenicity, calcification, intranodular blood flow, cervical lymph node, and tumor node metastasis (TNM) stage were compared between MTC and PTC groups. Age, sex, involvement of the thyroid gland, margin, and calcification were similar for the MTC and PTC groups. Compared with the PTC group, the lesion size in the MTC group was significantly larger (*P* < 0.001). A taller-than-wide shape (aspect ratio > 1) was significantly less likely in the MTC group than the PTC group (*P* < 0.001). A mixed echogenicity was significantly more common in the MTC group than the PTC group (*P* = 0.003). The MTC group had significantly enhanced intranodular blood flow (*P* < 0.001). The TNM stage of the MTC group was significantly higher than that of PTC group (*P* = 0.001). Medullary thyroid carcinomas differ significantly from PTCs in lesion size, shape, echogenicity, and intranodular blood flow.

## INTRODUCTION

Medullary thyroid carcinoma (MTC) originates from calcitonin-secreting parafollicular cells of the thyroid and accounts for 3.5-10% of all thyroid malignancies. Medullary thyroid carcinoma is characterized by early lymphatic metastatic spread and accounts for 13% of all thyroid cancer-related deaths [1]. Both sporadic and hereditary forms of MTC exist [2]. Hereditary MTC is an autosomal dominant genetic disease with the RET mutation of the proto-oncogene [3] and is a subtype of multiple endocrine neoplasia type 2 (MEN 2). Other subtypes of MEN2 include MEN2A (MTC combined with pheochromocytoma and hyperparathyroidism) and MEN2B (MTC combined with pheochromocytoma, multiple mucosal neuroma, and Marfan syndrome). Patients with only a family history of MTC are described as having familial medullary thyroid carcinoma [4]. Medullary thyroid carcinoma shows early lymph node metastases, aggressive invasion of the surrounding vital organs, insensitivity to radiation therapy and chemotherapy, and poor prognosis. Unilateral or bilateral thyroid tumors are the most common clinical features.

Treatment of cancer is comprehensive and based on surgery, mainly including preoperative evaluation, surgical selection, and lymph node dissection. Hereditary MTC has a higher proportion of multifocal lesions than sporadic MTC, so patients with hereditary medullary thyroid carcinoma undergo bilateral thyroidectomy as a matter of course. Early detection and preventive surgery are very important in the treatment of MTC. Most research has focused on the sonographic features of papillary thyroid carcinomas (PTCs), which account for more than 70% of all malignant thyroid tumors [5–8]. Compared with benign nodules, MTC and PTC show malignant nodule features, including ill-defined margin, irregular shape, hypoechoic echogenicity, solid nodules, and calcification (mainly microcalcifications) [9].

To our knowledge, only a few studies have compared ultrasonic features of MTC and PTC to date [10, 11]. Postoperative stages were judged by pathological specimens using the sixth edition of thyroid cancer staging criteria published by the Union for International Cancer Control [12]. Detection of RET gene mutation is routinely recommended for all patients with MTC and their first-degree relatives. However, detection of RET gene mutation has only been performed for a few patients in the biological chip center of Yantai Yuhuangding hospital, owing to the cost of this test, which is not covered by medical insurance. Although some cases of MTC can be assessed initially through family history and biochemical indicators, doctors must evaluate the nodules by ultrasonography under conditions of unknown clinical information. Kim et al. [11] indicate that lesion shape, as determined by ultrasonography, is different between MTC and PTC. Lee et al. [10] show that lesion size, cystic changes, and echogenicity are different between MTC and PTC. In this study, we collected clinical and ultrasonic data from northern Chinese mainland residents, and compared patients’ age and sex, unilateral or bilateral involvement of the thyroid gland, abnormal cervical lymph node, intranodular blood flow, and TNM stage.

## RESULTS

All 35 MTC patients and 96 PTC patients were surgically and pathologically confirmed at Yantai Yuhuangding hospital. Of 35 MTC patients, 27 (77.1%) were sporadic; one of the sporadic MTC cases also had minor PTC. The remaining eight cases (22.9%) were hereditary. Seven of the hereditary MTC cases were MEN 2A or MEN 2B; one case was familial medullary thyroid carcinoma. Comparisons of age, sex, glands involvement, and TNM stage are shown in Table 1. The general situation of the two groups in age (*P* = 0.399), sex (*P* = 0.126), gland involvement (single and double) (*P* = 0.109) showed no statistical significance difference. The TNM stage of the MTC group was significantly higher than that of the PTC group (*P* = 0.001) (Table 1). Preoperative serological test results of some of the MTC patients are shown in Table 2.

**Table 1:** Comparative analysis of the clinical characteristics of MTC and PTC patients

	MTC (*n* = 35)	PTC (*n* = 96)	*P* value
Gender			0.126
Male	19 (54.3%)	38 (39.6%)	
Female	16 (45.7%)	58 (60.4%)	
mean age	46.2 ± 11.5	44.5 ± 10.5	0.399
Gland involvement			0.109
Unilateral	27 (61.4%)	87 (73.1%)	
Bilateral	17 (38.6%)	32 (26.9%)	
TNM stage			0.001
I	8 (22.9%)	59 (61.5%)	
II	5 (14.3%)	7 (7.3%)	
III	13 (37.1%)	15 (15.6%)	
IV	9 (25.7%)	15 (15.6%)	

MTC, medullary thyroid carcinoma; PTC, papillary thyroid carcinoma.

**Table 2:** Preoperational serological test results of the MTC patients

Case	Serum calcitonin (2.93-34.8ng/l)	Carcinoembryonic antigen (CEA) (<5.0ng/ml)
1	>1535	198.2
2	no	66.28
3	no	5.62
4	no	40
5	>1535	37.55
6	no	3.61
7	69.4	72.55
8	865.02	4.90
9	55.65	5.70
10	71.17	9.74
11	no	143.0
12	296.65	283.56
13	33.13	27.9
14	33.94	36.5
15	no	56.44
16	521.28	59.78
17	1460	52.37
18	1251.75	12.28
19	1375.8	19.5
20	494.4	5.55

MTC, medullary thyroid carcinoma; no, data missing.

**Figure 1:**
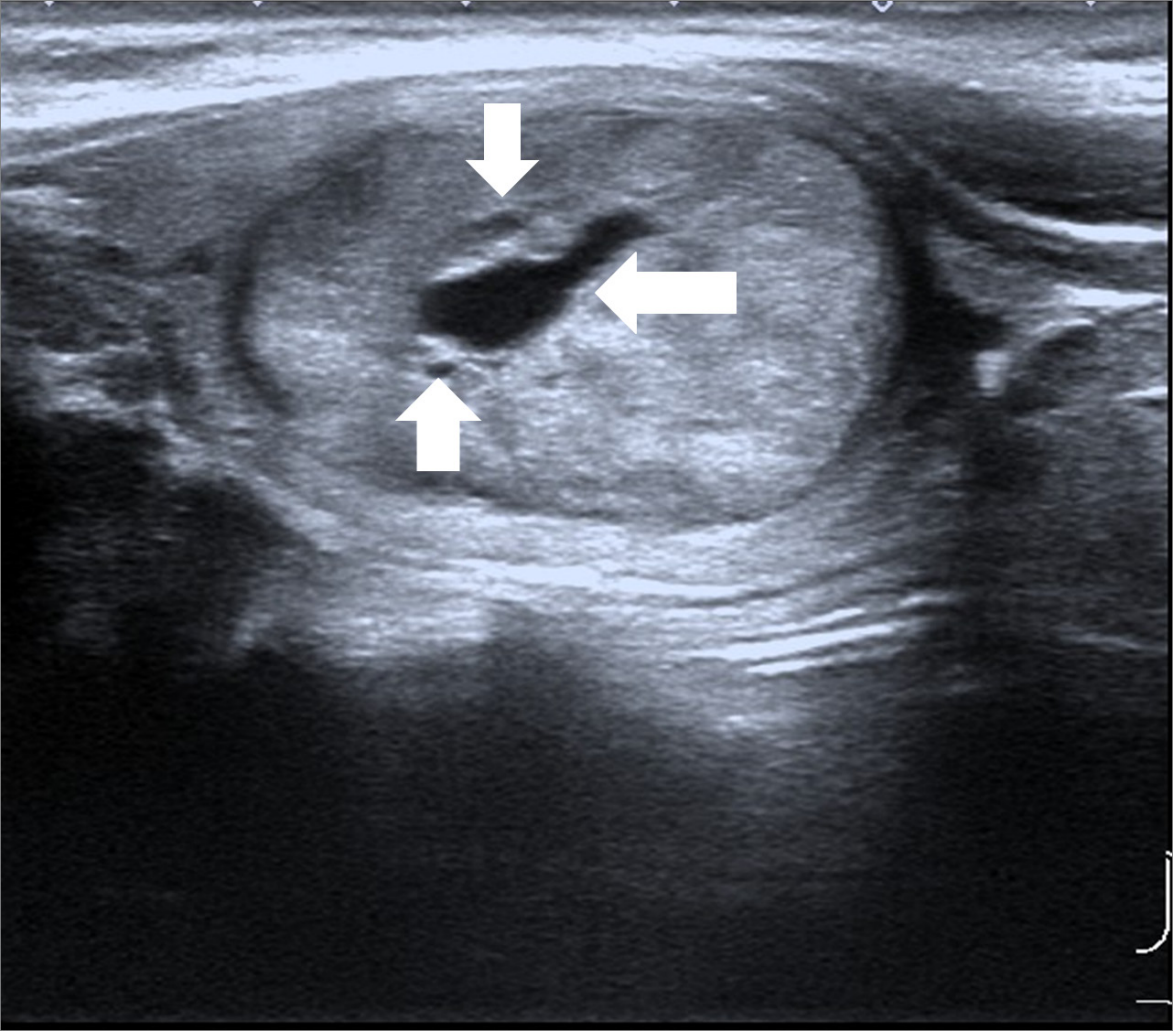
Male patient, 33 years old, MEN 2 MTC Lesion was oval in shape, with well-defined margin, mixed echogenicity, some cystic changes (arrows).

**Figure 2:**
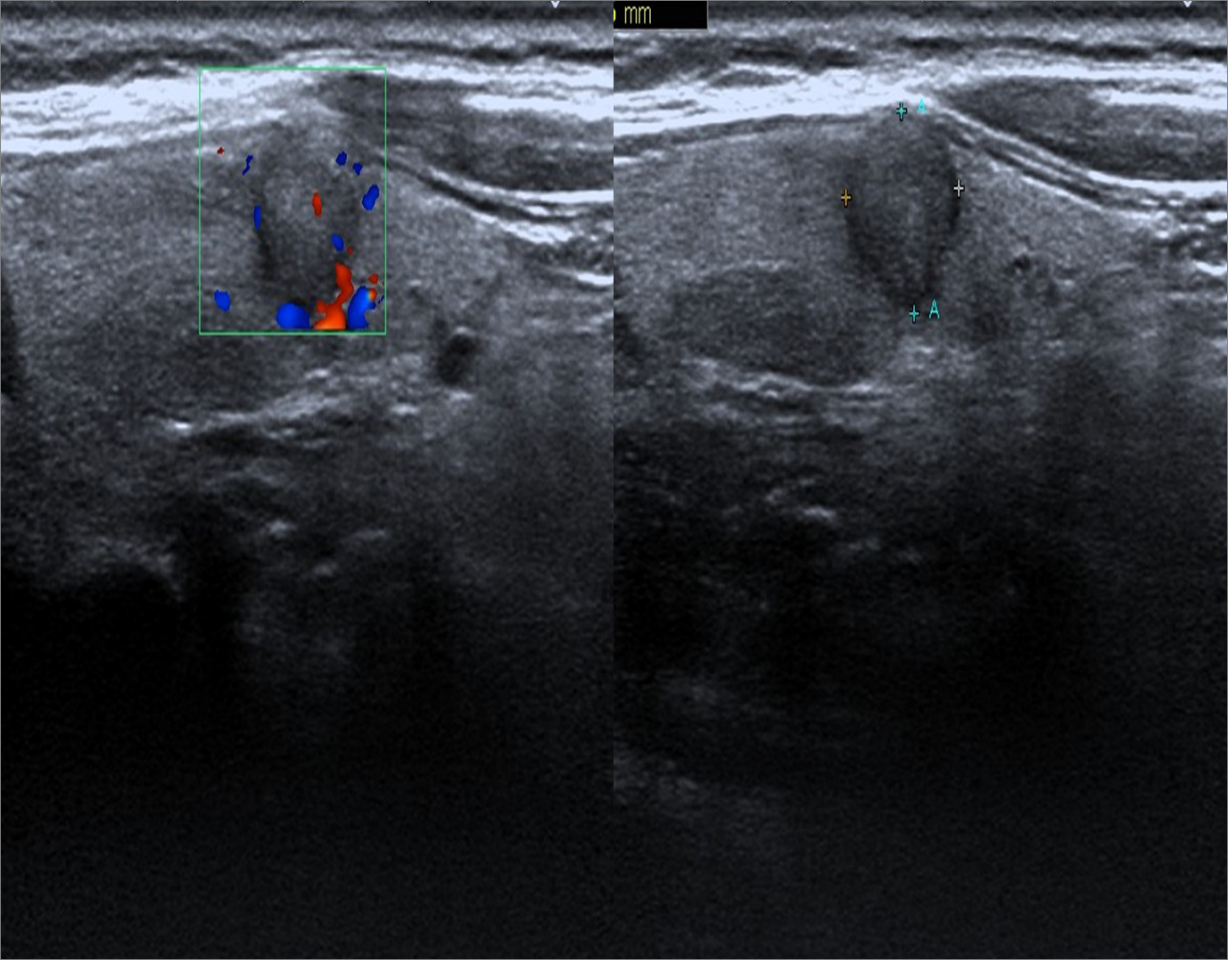
Female patient, 52 years old, PTC Lesions had aspect ratio > 1, ill-defined margin, hypoechogenicity, slightly visible intranodular blood flow.

**Figure 3:**
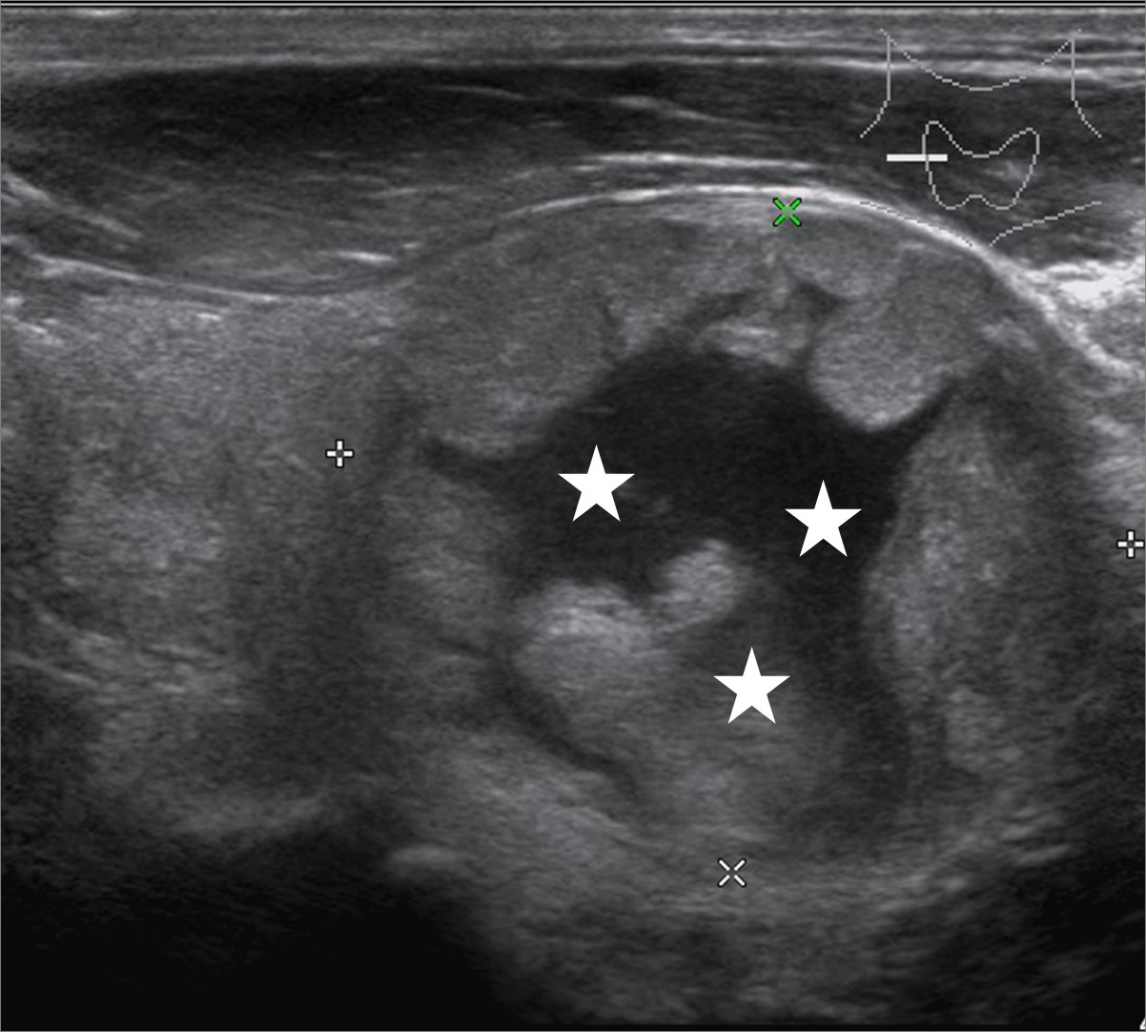
Male patient, 62 years old, sporadic MTC Lesions showed mixed echogenicity, more cystic components (*).

**Figure 4:**
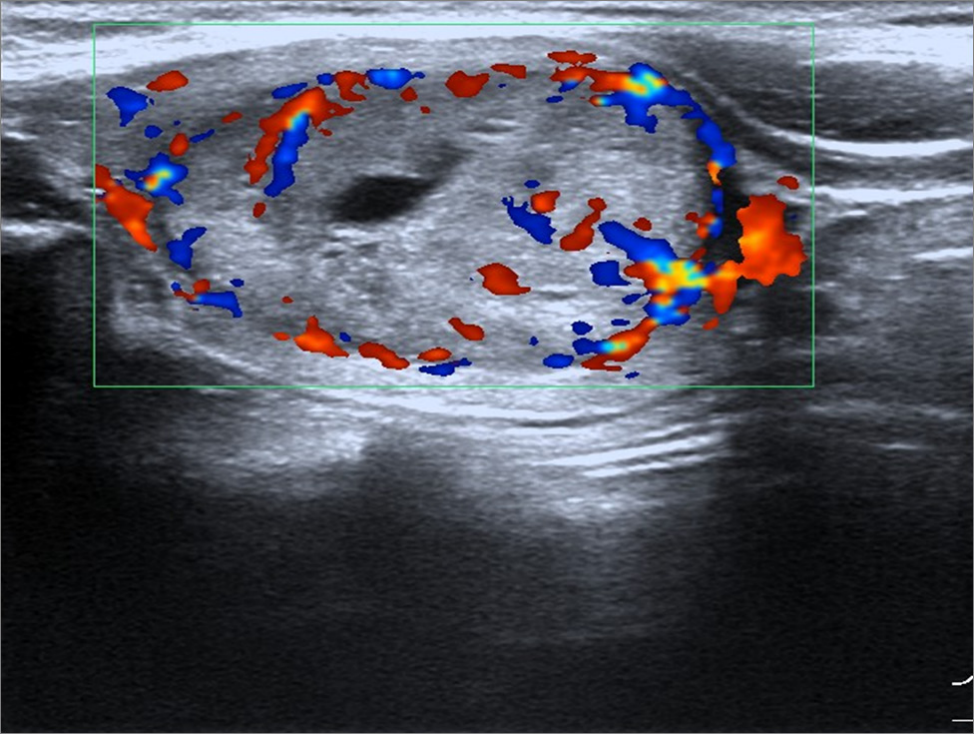
Female patient, 35 years old, sporadic MTC Lesion had aspect ratio < 1, enhanced blood flow.

Specific ultrasound characteristics of 44 MTCs and 119 PTCs are shown in Table 3. Like PTC, MTC showed ultrasonographic features of malignant nodules, including marked hypoechogenicity (37/44, 84.1%), ill-defined margin (28/44, 63.6%), microcalcifications (24/44, 54.5%), and abnormal cervical lymph node (16/35, 45.7%). However, compared with PTC, MTC had significantly larger lesion size (2.10 ± 1.70 cm *vs*. 0.80 ± 0.70 cm, *P* < 0.001). Most of the lesions in MTC were oval shaped (Figure 1). The MTC group only had three lesions (3/44, 6.8%) with aspect ratio > 1, which was significantly less than the PTC group (68/119, 57.1%, *P* < 0.001) (Figure 2). The MTC group had seven lesions (7/44, 15.9%) with a mixed echogenicity (Figures 1 and 3); this was significantly higher than the PTC group (3/119, 2.5%, *P* = 0.003). The MTC group had 40 lesions with enhanced blood flow signals (40/44, 90.9%) (Figure 4), which was significantly higher than the PTC group (47/119, 39.5%, *P* < 0.001). Both MTC and PTC groups showed abnormal cervical lymph nodes; the MTC group had 16 cases (16/35, 45.7%) and the PTC group had 32 cases (32/96, 33.3%). The difference between the two groups was not statistically significant (*P* = 0.21).

**Table 3:** Comparative analysis of ultrasound characteristics of MTC and PTC

	MTC (*n* =44)	PTC (*n* =119)	Statistics	*p* value
Lesion size (cm)M,Q	2.10,1.70	0.80,0.70	Z=6.70	<0.001
Shape			χ^2^=38.40	<0.001
Aspect ratio >1	3 (6.8%)	68 (57.1%)		
Aspect ratio≤1	41 (93.2%)	51 (42.9%)		
Margin			χ^2^=3.60	0.055
Well-defined	16 (36.4%)	26 (21.8%)		
Ill-defined	28 (63.6%)	93 (78.2%)		
Echogenicity			Fisher	0.003
Hypoechoic	37 (84.1%)	111 (93.3%)		
Isoechoic/hyperechoic	0	5 (4.2%)		
Mixed	7 (15.9%)	3 (2.5%)		
Calcifications			Fisher	0.488
Microcalcifications	24 (54.5%)	63 (52.9%)		
Macrocalcifications	1 (2.3%)	8 (6.7%)		
Rim calcifications	0	1 (0.8%)		
No calcifications	19 (43.2%)	47 (9.5%)		
Blood flow			χ^2^=40.84	<0.001
Enhanced	40 (90.9%)	47 (9.5%)		
Presence	2 (4.5%)	59 (49.6%)		
Absence	2 (4.5%)	13 (10.9%)		
Cervical lymph node			χ^2^=1.71	0.21
Abnormal	16/35 (45.7%)	32/96 (33.3%)		
Normal	19/35 (54.3%)	64/96 (66.7%)		

MTC, medullary thyroid carcinoma; PTC, papillary thyroid carcinoma.

## DISCUSSION

Our study indicates that there is no significant difference in age, sex, unilateral, or bilateral thyroid gland involvement and calcification between the MTC and PTC groups. However, the MTC group significantly differs from the PTC group in lesion size, shape, echogenicity, and blood flow. Moreover, serum calcitonin and carcino-embryonic antigen tests can be used for MTC screening.

Medullary thyroid carcinoma is a rare neuroendocrine tumor that originates from the parafollicular cells of the thyroid gland. These tumor cells can synthesize a variety of biological substances, such as calcitonin, carcino-embryonic antigen, and calcitonin gene-related peptide. Elevated calcitonin and carcino-embryonic antigen levels are the most significant clinical features of MTC; hence, calcitonin is used as a sensitive biochemical marker of MTC [13]. Elevated serum calcitonin levels result from its overproduction from calcitonin-secreting parafollicular cells of the thyroid. Some researchers believe that the detection of serum calcitonin is more sensitive than fine needle aspiration [14]. In this study, only a minority of patients in whom ultrasonic inspection detected suspicious nodules were examined preoperatively with fine needle aspiration. In our study, the serum calcitonin positive rate in the MTC group was 100%, and the carcino-embryonic antigen positive rate was 92.8%. Therefore, serum calcitonin and carcino-embryonic antigen blood tests are necessary in patients with suspected MTC.

Woliński et al. [15] indicate that MTC occurs in older patients ( > 50 years), is more common in women than men, and is typically sporadic. In our study, the mean age of the 35 MTC patients was 46.2 ± 11.5 years; the group comprised 19 men and 16 women. Seven of the patients with MTC reported at the clinic with paroxysmal headache, intermittent abdominal pain with headache, pallor, palpitations, and high blood pressure. One of the patients with MTC reported at the clinic because of a family history of MTC. Therefore, sex, age, MEN family history, and clinical symptoms are not reliable for MTC screening. From the lesion margin aspect, most studies [11, 16] show that MTC has a well-defined margin, in comparison with PTC. However, our findings show that there is no significant difference between these two groups.

Our study indicates that the lesion size in the MTC group (median, 2.1 cm; interquartile, 1.7 cm) was significantly greater than the PTC group (median, 0.8 cm; interquartile, 0.7 cm). Although the MTC group had larger lesions, 35% of MTC cases have metastases at the time of initial diagnosis [17], indicating the occult characteristics of the MTC.

We found that lesion shape is different between MTC and PTC groups. Lesions with an aspect ratio > 1 only accounted for 6.8% of all lesions in the MTC group, which is significantly less than the PTC group (57.1%). Our results are similar to those of Woliński [15], in which lesions with aspect ratio > 1 only accounted for 14.4% of 157 MTC cases.

Both MTC and PTC groups showed hypoechoic echogenicity in our study. However, five (5/119, 4.2%) lesions in the PTC group showed hyperechoic echogenicity, whereas the MTC group did not have any hyperechoic lesions. These results are consistent with a previous study [15], in which hyperechoic lesions were absent in 157 cases of MTC. Interestingly, we found that the percentage of the mixed echogenicity in MTC and PTC groups was significantly different; 7 of 44 lesions (15.9%) in the MTC group had mixed echogenicity, while 3 of 119 lesions (2.5%) in the PTC group had mixed echogenicity. Some studies [18] have suggested that mixed echogenicity (cystic component > 50%), accompanied with honeycomb change, is an indication of benign nodules. However, our results indicated that the mixed echogenicity of thyroid nodules can also be an ultrasonic feature of MTC.

In our study, about half the lesions in MTC and PTC groups had visible microcalcifications, and there was no significant difference between these two groups (*P* = 0.488). Most of the lesions in the MTC group had enhanced blood flow signals (40/44, 90.9%), which was significantly higher than for the PTC group (47/119, 39.5%, *P* < 0.001). Saller et al. [19] suggest that grayscale ultrasound is an important means of thyroid nodule examination, and state that detection of intranodular blood flow by color Doppler sonography provides no additional information for the diagnosis of MTC or other malignant nodules. The intensity of ultrasonic blood flow signals is related to a variety of factors, such as tumor size and tumor-specific pathological type. Foschini et al. [20] found that the vascular composition of MTC is similar to that of the differentiated mast cell tumors, in which the peripheral blood vessels invade and differentiate into smaller vessels.

Gimm et al. indicate that the incidence of central group lymph node metastasis at initial surgery of MTC is 75-80% [21]. In this study, 22 patients had confirmed pathological lymph node metastasis (22/35, 62.9%); however, preoperative ultrasound examination only found lymph node abnormalities in 16 cases (16/35, 45.7%), indicating that the use of ultrasound for diagnosis of cervical lymph nodes abnormality has limitations [22]. Guerrero et al. [23] reported that 22% of MTC patients made a first clinical visit in response to neck pain (defined as neck discomfort, pain, throbbing, hoarseness, difficulty swallowing, etc.). Patients with MTC with neck pain had increased lymph node metastasis risk (82%). In our study, we found that three cases (3/35, 8.6%) had symptoms of neck pain and two of these (2/3, 66.7%) had confirmed lymph node metastasis. There were 32 cases without neck pain; 20 of these (20/32, 62.5%) had pathologically confirmed lymph node metastasis.

Kim et al. [11] found that the only difference between MTC (21 cases) and PTC (114 cases) was that MTC has more circular and elliptical lesions (57.1% *vs* 25.4%). Lee et al. [10] reported that lesion size (2.29 ± 1. 53 *vs* 1.10 ± 0.71), cystic changes (32.6% *vs* 3.6%) and the solid part of the echogenicity (58.7% *vs* 30.9%) are significantly different between MTC (42 cases) and PTC (51 cases) groups.

In conclusion, there is no statistical difference in sex, age, or unilateral or bilateral thyroid gland involvement between MTC and PTC groups in our study. Both MTC and PTC have the common ultrasound characteristic of malignant nodules, including ill-defined margin, hypoechoic echogenicity, microcalcifications, and cervical lymph node abnormalities. However, there were significant differences in lesion size, shape, echogenicity, and blood flow signal between the MTC and PTC groups. The TNM stage of the MTC group was significantly higher than that of the PTC group. Compared with the PTC group, the lesions in MTC groups were significantly larger; in addition, there were fewer lesions with an aspect ratio > 1, more cystic changes, and enhanced blood flow. In addition, serum calcitonin and carcino-embryonic antigen tests are important in the diagnosis of MTC, which can be combined with genetic testing if necessary.

## MATERIALS AND METHODS

### Patients

This study included 44 MTC cases (35 patients) that were surgically and pathologically diagnosed from July 2001 to July 2014, and 119 PTC cases (96 patients) that were surgically and pathologically diagnosed from July 2012 to July 2014, in Yantai Yuhuangding hospital. Age, sex, lesion location, and lesion sonographic characteristics were compared between MTC and PTC groups. The inclusion criteria were as follows: the patient received preoperative ultrasound examination in our hospital and ultrasound data were complete; the patient received surgery in our hospital; the pathology was confirmed as MTC or PTC. The exclusion criteria were as follows: the patient with MTC received surgery in another hospital and underwent further surgery in our hospital. This study was approved by the hospital ethics committee. Informed consent has been obtained from all participants.

### Sonography

High-resolution ultrasound examinations of the thyroid were performed in all patients with GELogiq9 (New York), GEE8 (Seoul, Korea), or Philips IU22 (New York) scanners equipped with a 5-12 MHz linear array transducer. The settings were set for thyroid or superficial organ examination and the color gain was adjusted to minimize background noise. All ultrasound examinations were performed by radiologists with more than 5 years of experience in thyroid imaging. Each patient's age and sex were recorded before examination. Each patient was in a supine position during examination.

Anterior multi-plane scanning was performed to determine the size of the thyroid, echogenicity, the presence of nodules, unilateral or bilateral gland involvement, nodule location, number, size, margin, shape, echogenicity, internal content, calcification, blood flow, and cervical lymph node. Some patients received preoperative serum calcitonin and carcino-embryonic antigen tests.

### Imaging analysis

Echogenicity was defined as markedly hypoechoic (equal to or less than the strap muscle echogenicity), hypoechoic (ranging between the strap muscle and the thyroid echogenicity), isoechoic (equal to the thyroid echogenicity), or hyperechoic (greater than the thyroid echogenicity). Lesions containing cystic change were defined as showing mixed echogenicity. The margins were recorded as well-defined or ill-defined. The aspect ratio was recorded as either > 1 (the anteroposterior dimension was longer than the transverse diameter) or < 1 (the anteroposterior dimension was less than the transverse diameter), according to the ultrasound description. Calcifications were defined as macrocalcifications (large bright spots, larger than 2 mm in diameter, presence of acoustic shadowing) or microcalcifications (multiple punctuate bright spots, less than 2 mm in diameter, hyperechogenicities, absence of acoustic shadowing). If macrocalcifications coexisted with microcalcifications, these were classified as microcalcifications. The intranodular blood flow was recorded as “no blood flow signal,” “visible blood flow,” or “enhanced blood flow signal.” With reference to Koike et al. [24], rich blood flow of color Doppler manifestation of thyroid nodules was identified as rod-like, strip shaped, penetration of color Doppler signals, or more than five points of blood flow signals within the nodule. Abnormal cervical lymph node was diagnosed if the short axis diameter was > 0.5 cm with disappearance of hyperechoic structure, accompanied by any of the following signs: uneven internal echogenicity, irregular blood flow, calcification, or cystic changes.

### Statistical analysis

SPSS17.0 software was used for statistical analysis. Comparison of age was analyzed using an independent-sample *t* test; a Wilcoxon rank sum test was used for tumor size comparison. Sex, and sonographic features, such as: unilateral or bilateral thyroid involvement, margin, echogenicity, internal components, calcification, blood flow, and shape were compared using Pearson's chi-squared test or Fisher's exact test. The TNM stages were combined into two classifications and a chi-square test was performed. Statistical significance was defined for *P* < 0.05.
